# Skin Vaccination against Rotavirus Using Microneedles: Proof of Concept in Gnotobiotic Piglets

**DOI:** 10.1371/journal.pone.0166038

**Published:** 2016-11-08

**Authors:** Yuhuan Wang, Anastasia Vlasova, Daniel E. Velasquez, Linda J. Saif, Sukumar Kandasamy, Efrat Kochba, Yotam Levin, Baoming Jiang

**Affiliations:** 1 Gastroenteritis and Respiratory Viruses Laboratory Branch Division of Viral Diseases, Centers for Disease Control and Prevention, Atlanta, Georgia, United States of America; 2 Food Animal Health Research Program, Ohio Agricultural Research & Development Center, The Ohio State University, Wooster, Ohio, United States of America; 3 NanoPass Technologies Ltd., Nes Ziona, Israel; University College Cork, IRELAND

## Abstract

Live-attenuated oral rotavirus (RV) vaccines have lower efficacy in low income countries, and additionally are associated with a rare but severe adverse event, intussusception. We have been pursuing the development of an inactivated rotavirus vaccine (IRV) using the human rotavirus strain CDC-9 (G1P[8]) through parenteral immunization and previously demonstrated dose sparing and enhanced immunogenicity of intradermal (ID) unadjuvanted IRV using a coated microneedle patch in comparison with intramuscular (IM) administration in mice. The aim of this study was to evaluate the immune response and protection against RV infection and diarrhea conferred by the administration of the ID unadjuvanted IRV using the microneedle device MicronJet600^®^ in neonatal gnotobiotic (Gn) piglets challenged with virulent Wa G1P[8] human RV. Three doses of 5 μg IRV when administered intradermally and 5 μg IRV formulated with aluminum hydroxide [Al(OH)_3_] when administered intramuscularly induced comparable rotavirus-specific antibody titers of IgA, IgG, IgG avidity index and neutralizing activity in sera of neonatal piglets. Both IRV vaccination regimens protected against RV antigen shedding in stools, and reduced the cumulative diarrhea scores in the piglets. This study demonstrated that the ID and IM administrations of IRV are immunogenic and protective against RV-induced diarrhea in neonatal piglets. Our findings highlight the potential value of an adjuvant sparing effect of the IRV ID delivery route.

## Introduction

Rotavirus (RV) infection causes severe dehydrating diarrhea in young children under 5 years of age worldwide. In 2011 the annual estimated number of RV disease-associated death in the <5 year old was 192,700 (133,100–284,400) and the majority of the fatalities occur in low income countries of Africa and Asia where healthcare is not readily available or accessible [1]. Two live oral vaccines, Rotarix and RotaTeq, have been shown to be generally safe and efficacious in developed and middle-income countries, and have been licensed for use in more than 100 countries, including the introduction into routine national immunization programs in 81 countries [2]. However, these vaccines have been shown to be less efficacious in many low-income countries where an effective vaccine is needed most due to high morbidity and mortality [3–7]. The mechanisms for the gradient efficacies among children in different countries are likely to be multifactorial, including in part the frailty of health care systems. Over the last several years, various intervention studies, such as transient withholding of breastfeeding at the time of immunization, delayed administration or addition of a third or more dose of vaccine, have been conducted, but none to date have shown real improvement in the performance of the vaccines [8–11]. The two vaccines have also been shown to be associated with rare but severe intussusception in infants who received vaccine [12]. In addition, when these vaccine virus strains and wild type human rotaviruses are present in the gut, they can reassort to produce new strains, including virulent double bovine-human rotavirus reassortants [13–16].

To address the problems associated with live oral rotavirus vaccines, parenteral immunization with inactivated rotavirus vaccine (IRV) is an attractive approach for protection of children against RV disease. Early studies provided the proof of principle of establishing protection by a live or inactivated animal RV, or virus-like particles via intramuscular (IM) administration [17–19]. After that we developed a candidate human RV vaccine CDC-9 (G1P[8]) and demonstrated that this thermally inactivated CDC-9 formulated with Al(OH)_3_ adjuvant and administered by IM injection was highly immunogenic in mice and guinea pigs and conferred protection against homologous rotavirus challenge in gnotobiotic (Gn) piglets [20–22]. Thus, a safer and potentially more widely effective IRV could be an alternative to the prevention of rotavirus disease. However, the cost to manufacture an IRV for parenteral vaccination may be higher than that of producing live oral vaccines due to extra processes for purification and inactivation.

One way to reduce the cost of the IRV is to deliver a fraction of the IM dose via intradermal (ID) vaccination using novel innovative microneedle devices. The skin is rich in antigen presenting cells (Langerhans cells, dermal dendritic cells, macrophages) and ID vaccination has been shown to mount potent immune responses. Smallpox, tuberculosis and rabies vaccines administered via ID route were highly effective in the prevention of these bacterial and viral diseases [23–25]. Recent studies have demonstrated that inactivated polio vaccine (IPV) given at a fractional dose versus full IM dose by ID route using devices such as needle-free jet injector and hollow microneedles, induced seroconversions comparable to that of a full IM-dose IPV [26]. Similarly, inactivated influenza vaccine, Fluzone, when administered using an ID device at 60% of its IM vaccine dose, has shown equivalent protective efficacy against seasonal influenza [27, 28]. MicronJet600^®^, a device registered by the US FDA, has been successfully tested to deliver IPV, influenza, live attenuated zoster and other vaccines in clinical trials [26, 29–31]. In addition, significant dose sparing, using 20% of the dose, was achieved with various influenza vaccines with the MicronJet600® device [28, 29]. Furthermore, we recently demonstrated apparent dose sparing and enhanced immunogenicity of the IRV without adjuvant when administered transcutaneously using a coated metal microneedle patch compared to IM administered IRV in mice [32].

The physiology and immune responses of Gn pigs mimic those of humans. Gn piglets also show clinical signs of diarrhea and virus shedding post-HRV challenge similar to infants. Moreover being outbred, pigs exhibit heterogeneity in immune responses similar to humans. Thus, neonatal Gn piglets are a relevant animal model to investigate IRV [33–35]. In this report, we assessed the immune responses of IRV administered by two different ways: ID using a hollow microneedle injection device MicronJet600^®^ without adjuvant and IM with Al(OH)_3_ as an adjuvant; and also evaluated the protection conferred by both administrations against RV-induced diarrhea in neonatal Gn piglets challenged with the most prevalent human RV strain, Wa (G1P[8]).

## Materials and Methods

### Ethics statement

The study protocol was approved by Institutional Animal Care and Use Committee (IACUC) of the Ohio State University (Protocol Number: 2010A00000088) (S1 File). All the piglets were maintained, samples collected, and then euthanized, and all efforts were made to minimize the suffering of animals. The IACUC protocol describes early removal criteria (ERC) and humane euthanasia methods. The ERC include euthanasia of animals if they developed severe diarrhea or were non-responsive to treatment. Other criteria used to determine the end of study include depression, anorexia, lethargy, lack of weight gain by visual appearance, abnormal behavior, and dehydration. However, no animal developed such symptoms or became severely ill during this experiment. No animals died prior to the experiment termination. Prior to euthanasia by electrocution the pigs were anesthetized using TKZ combo (Telazol 100mg/ml, Ketamine 100mg/ml, Xylazine 100mg/ml). Otherwise the experiment involved no suffering to the animals and no modifications to housing/feeding/handling procedures (described in the protocol) were required. Piglets were euthanized at the study termination.

### Virus inocula

The intestinal contents of virulent human RV group A strain Wa (VirWa) G1P[8] infected Gn pigs were diluted in minimal essential medium (MEM; Invitrogen, Carlsbad, USA) and used for challenge at a dose of 5x10^5^ fluorescent-forming units (FFU). The 50% infectious dose (ID50) of VirWa HRV in pigs was determined as approximately 1 FFU [36]

### Vaccine preparation

CDC-9, a human G1P[8] rotavirus strain, was cultivated in Vero cells using Iscove’s Modified Dulbecco’s Medium (IMDM; Invitrogen, Grand Island, NY) [37]. Triple-layered particles (TLPs) were purified from culture supernatants by CsCl gradient equilibrium ultracentrifugation, suspended in Hanks balanced salt solution (HBSS) containing 10% sorbitol, and inactivated by incubation at 58°C for 4 hours [20]. Inactivation of CDC-9 was confirmed by the lack of virus growth in three sequential passages in Vero cells, which was measured with a commercial immunoassay (Premium® Rotaclone® Kit; Meridian Diagnostics, Cincinnati, OH). Each dose of ID vaccine contained 5 μg of IRV antigen in 0.2 ml. For IM vaccine, each dose contained 5 μg of IRV antigen formulated with 600 μg of Al(OH)_3_ in 1.0 ml; 89% of the IRV antigen was adsorbed to Al(OH)_3_ gel.

The MicronJet600^®^ device used for ID injection was manufactured by NanoPass Technologies [30, 31]. It is a single-use, microneedle based device, for ID delivery of vaccines and other molecules into the skin. The device consists of 3 microneedles, each 0.6 mm in length, on a hub, that can be mounted on any standard syringe. This device has the shortest needles ever registered with the FDA.

### Gnotobiotic pigs and experimental design

Gn piglets were derived by hysterectomy from the sows bred at the OSU swine herd. The pigs were maintained in plastic flexible positive pressure isolator units supplied with filtered air and received a UHT bovine milk diet (Parmalat) twice a day as described in the protocol. The lights stayed on from 7AM-7PM and were regulated by automatic turn on/off. For vaccination, eleven 3-day old Gn piglets from one litter were randomly divided into three groups; 3 received 2 x 100 μl of HBSS with 10% sorbitol by ID device as placebo control and 4 each received 2 x 100 μl of IRV by ID device or 2 x 500 μl of IRV adsorbed to Al(OH)_3_ gel by IM injection. ID administrations were given at two sites on the abdomen with rich draining lymph nodes. The skin was not shaved prior to the application. IM vaccine was administered in the ham area of each hind leg. Three doses of vaccine or placebo were inoculated on post-vaccination days 0, 10 and 21.

### Pig virus challenge, clinical observations and sample collection

On post-vaccination day 28, all 11 piglets were orally inoculated with the homologous human RV VirWa, one of the most prevalent RV strains worldwide at a dose of 5x10^5^ ID_50_. After Wa RV challenge, the animals were observed daily for diarrhea and other clinical signs from 0 to 10 post inoculation days (PID). To estimate the severity of diarrhea, fecal consistency was scored by qualified technicians as follows: 0 = solid; 1 = pasty; 2 = semi-liquid (moderate diarrhea); 3 = liquid (severe diarrhea). A score of ≥ 2 is considered diarrhea. Before and after Wa HRV challenge, rectal swabs were collected daily to assess RV antigen shedding. Whole blood samples were collected by jugular venipuncture in BD Li-Heparin vacutainers immediately before each inoculation and on days 3, 5, 10 and 14 after the challenge and processed for plasma according to the manufacturer’s instructions.

### Porcine IgG, IgA ELISA

Rotavirus-specific IgG or IgA in sera of piglets was measured by using a modified enzyme immunoassay (EIA) [20]. Briefly, 96-well plates (Immulon 2; NalgeNunc, Rochester, NY) were coated with diluted rabbit hyperimmune serum to human rotavirus Wa at 4°C overnight. After washing, the plates were incubated at 37°C first with 5% skim milk in PBS (blotto), and then with supernatants of Wa rotavirus-infected MA104 cell cultures (~10^6^ FFU/ml) (36), followed by the addition of serially diluted porcine sera. Plates were further incubated sequentially with biotinylated anti-swine IgG (KPL, Gaithersburg, Maryland) or biotinylated goat anti-porcine IgA (Bethyl Labs, Montgomery, TX), followed by addition of ExtrAvidin^®^−peroxidase (Sigma-Aldrich, St. Louis, MO). The color of reactions was developed with substrate tetramethyl benzidine (TMB; Sigma-Aldrich) and stopped with 1N HCl. The optical density (OD) at 450 nm was measured with an EIA reader (MRX Revelation, Dynex Technologies, Chantilly, VA). Antibody titer in a serum specimen was defined as the reciprocal of the highest dilution that gave a mean OD greater than the cutoff value [3 standard deviations above the mean OD of the blotto wells-(blank wells with no serum)].

### Porcine IgG avidity assay

Rotavirus IgG avidity assay was performed by modifying the protocol of RV IgG EIA with the denaturant agent diethylamine (DEA) [38]. One dilution series started at 1:200 and was washed 6 times with 0.05% Tween 20-PBS, the other dilution series started at 1:20 and was washed 3 times for 5 min with 60 mM DEA in 0.05% Tween 20-PBS (pH 11.0) and 3 times with 0.05% Tween 20-PBS. End titer avidity index percentages (etAI%) were obtained using the formula etAI% = (end-titer DEA curve / end-titer wash buffer curve) X 100 [38].

### Virus neutralization assays

Neutralizing activity in porcine sera was measured against homotypic and heterotypic human rotavirus strains Wa (G1P[8]), WI61 (G9P[8]) and MW333 (G8P[4]), with a modified microneutralization assay [20]. Each strain was individually optimized to use 5,000 FFU for Wa; 600 FFU for WI61 and MW333. Neutralizing titer was defined as the reciprocal of the highest dilution that gave a greater than 70% reduction in the absorbance value compared to that in virus-only control wells.

### Porcine cytokines

Cytokines in porcine sera were measured with Swine Cytokine Magnetic 7-Plex Panel kit (Life technologies, Grand Island, NY) using Bio-Plex 200 (Bio-Rad, Hercules, CA) according to manufacturer’s instructions. Data collection and analysis were done using Bio-Plex Manager 5.0.

### RV antigen detection

Human RV shedding was detected in rectal swabs using Premier Rotaclone® according to manufacturer’s instructions.

### Statistical analysis

Fisher’s exact test was used to compare proportions of animals with diarrhea and RV antigen shedding among groups. The Kruskall-Wallis rank sum test (non-parametric) was used to compare days of onset and duration of diarrhea and RV antigen shedding, cumulative diarrhea scores and cumulative ODs of RV antigen shedding (area under the curve, AUC) among groups that were recorded. Negative samples were assigned an arbitrary Ab titer of 1 for the calculation of geometric mean titers (GMTs). Neutralizing and RV Ab titers were log10-transformed prior to statistical analysis. Differences in Ab and neutralization Ab titers among groups were evaluated by comparison of means using a 2-tailed t-test at different time-points post virus inoculation. Statistical significance was assessed at p < 0.05 for all comparisons. Statistical analyses were conducted using IBM® SPSS® Statistics Version 21.

## Results

We assessed the safety of IRV in piglets by daily observation following each vaccination. No local reactogenicity at injection sites and no fever or other systemic adverse events were found in all placebo or IRV-vaccinated piglets.

### ID and IM-administered IRV induced potent IgA, IgG, IgG avidity and neutralizing antibody responses in piglets

**Fig 1:**
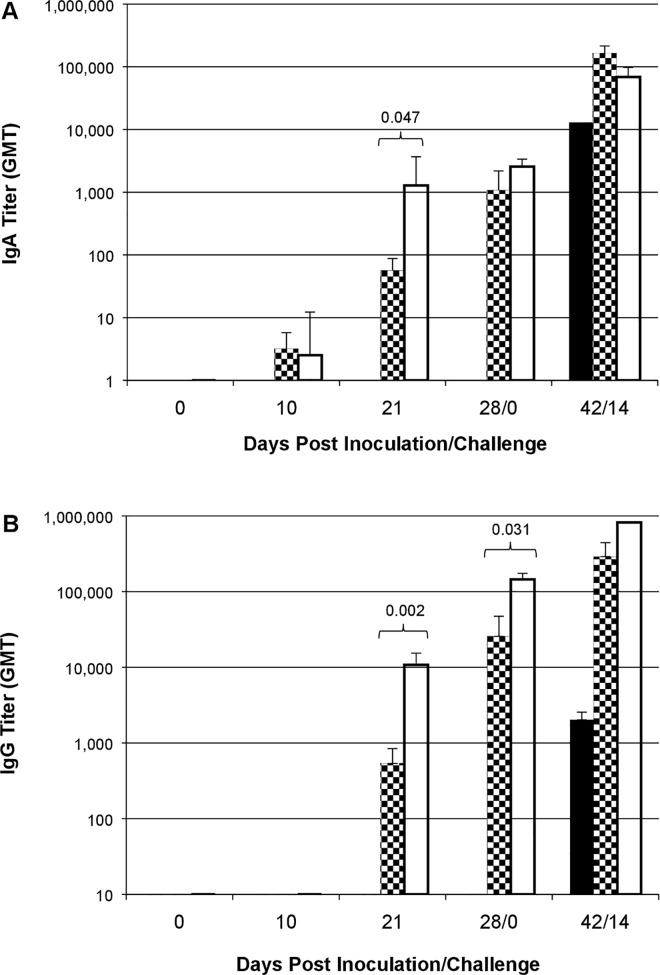
Rotavirus-specific serum IgA and IgG antibody titers in control, ID or IM IRV-vaccinated piglets. Each serum specimen was tested at an initial dilution of 1:10 for IgA (A) and 1:100 for IgG (B). If IgA or IgG activity was not detected at initial dilution, a value of 1 for IgA and 10 for IgG was used for calculation and graphic illustration. Data are presented as geometric mean titer (GMT) + one standard error (error bar). Filled, checker board and open bars represent control, ID and IM groups, respectively. Significant differences are indicated.

**Fig 2:**
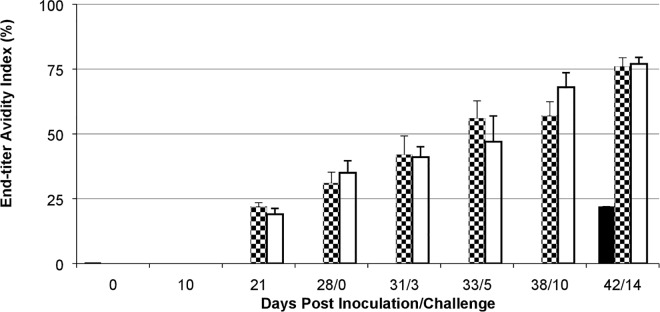
Avidity of rotavirus-specific IgG antibody in sera of control, ID or IM IRV-vaccinated piglets. Shown are IgG end-titer avidity index percentages (etAI%). Data are presented as mean + one standard error (error bar). Filled, checker board and open bars represent control, ID and IM groups, respectively.

We examined and demonstrated a dose-dependent rotavirus-specific IgA and IgG response to IRV without Al(OH)_3_ administered with an ID device or IRV formulated with Al(OH)_3_ administered by IM injection (Fig 1). Piglets developed low titers of IgA and no detectable IgG (< 100 titer) in sera by day 10 following first ID or IM vaccination. IM administration induced 19- and 20-fold higher geometric mean titers of IgA (p = 0.047) and IgG (p = 0.002) compared with ID vaccination by post-vaccination day 21 after the second dose. After three doses, ID and IM vaccines induced strong and comparable IgA (p = 0.252) and IgG (p = 0.031) titers at post-vaccination day 28. By contrast, piglets in control group had no detectable IgA and IgG titers in sera throughout the post-vaccination period of 28 days. To examine whether ID and IM vaccinations induced similar kinetics and accumulated strength of IgG interactions with the multiple antigenic epitopes in rotavirus, we measured sera for rotavirus-specific IgG avidity index (Fig 2). Piglets had no detectable levels of IgG avidity index in pre-bleed sera and sera 10 days post dose 1. IgG avidity index was seen at comparable levels in sera of ID and IM IRV-vaccinated piglets 11 days post dose 2 (21 days post-vaccination) (p = 0.317) and increased to a mean value of 31% and 35% seven days post dose 3 (28 days post-vaccination) (p = 0.502), respectively. IgG avidity index continued to increase to more than 70% in both ID and IM vaccinated animals 14 days after the oral challenge (p = 0.693). None of the piglets in control group had detectable IgG avidity index in sera throughout the study period until 14 days following the oral challenge.

**Fig 3:**
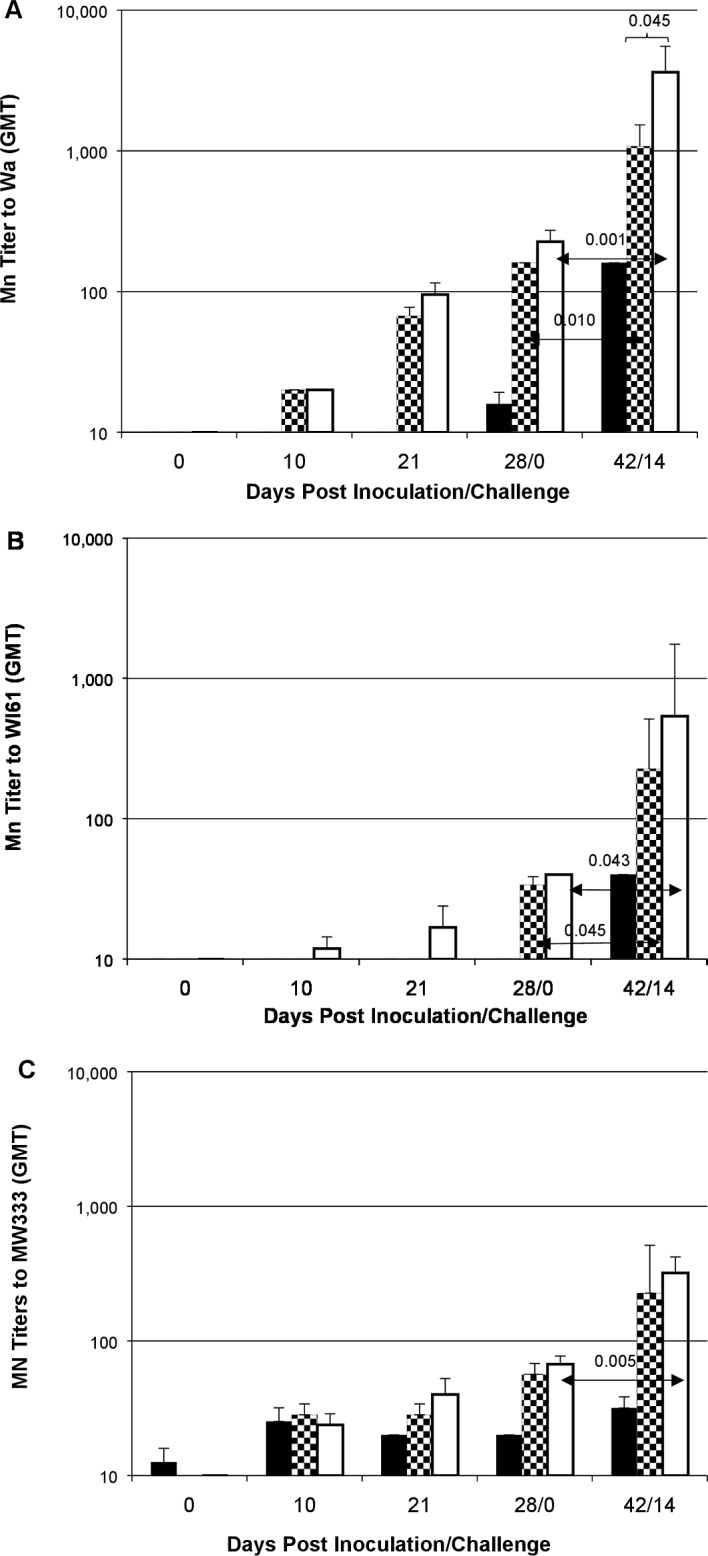
Neutralizing activity in sera of control, ID or IM IRV-vaccinated piglets. The neutralizing antibody titers to Wa (A), WI61 (B) and MW333 (C) strains were determined by neutralization assay. Each serum specimen was tested at an initial dilution of 1:20. For negative samples at 1:20 dilution, an arbitrary value 10 was used for calculation and graphic illustration. Data are presented as geometric mean titer (GMT) + one standard error (error bar). Filled, checker board and open bars represent control, ID and IM groups, respectively. Significant differences are indicated.

We also measured sera for neutralizing activity against homotypic and heterotypic human rotavirus strains (Fig 3). ID- and IM-administered IRV induced neutralizing antibody responses to the homotypic Wa strain; strong comparable titers were seen after two inoculations of ID and IM vaccinations (p = 0.207) and titers were further enhanced after three inoculations (p = 0.182). In addition, ID- and IM-administered IRV induced lower but number of inoculation-dependent neutralizing antibody responses to the partially heterotypic strain WI61 (G9P[8]) and the completely heterotypic strain MW333 (G8P[4]); comparable titers were seen after three IM injections and ID administrations (WI61: p = 0.391; MW333: p = 0.537). In contrast, piglets in control group had little or no detectable neutralizing antibody against homotypic and heterotypic strains throughout 28-day post vaccination. Oral challenge with a virulent Wa strain further boosted neutralizing antibody responses, as reflected by GMT to Wa by 16 folds (p = 0.001) and to WI61 and MW333 by 5–13 folds (p = 0.043, and p = 0.005 respectively) in IM vaccinated animals at post challenge day 14. Similarly, the oral challenge also boosted neutralizing antibody GMT to Wa by 6.7 fold (p = 0.010) and to MW333 and WI61 by 4–7 fold (p = 0.045, and p = 0.100 respectively) in ID vaccinated piglets at post challenge day 14.

### IRV vaccination modulated serum cytokine response to post virulent HRV challenge

**Fig 4:**
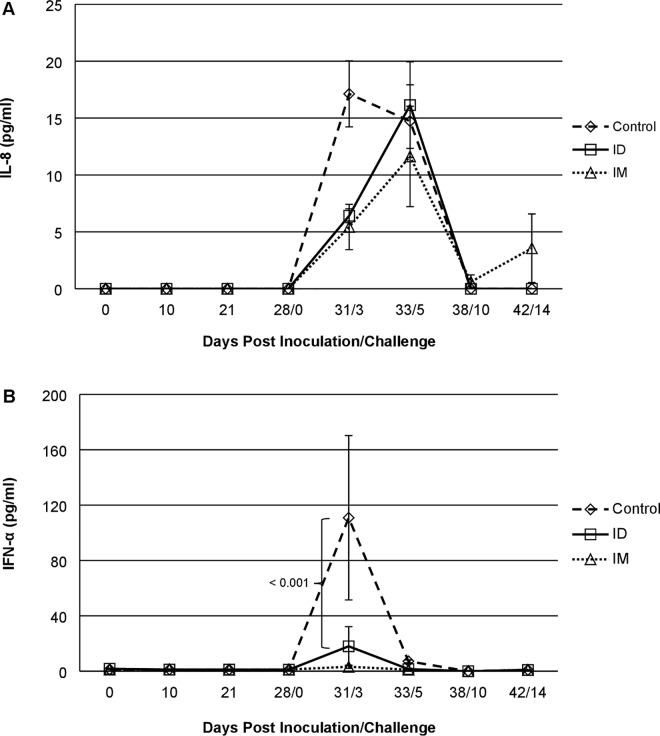
Kinetic profiles of cytokines in sera of control, ID or IM IRV-vaccinated piglets. Levels of IL-8 (A) and IFN-α (B) were measured with Swine Cytokine Magnetic 7-Plex Panel kit as described in the text. Levels of five other cytokines (IL-1β, IL-4, IL-10, IFN-ϒ, TNF-α) were not elevated (data not shown). Each data point denotes geometric mean concentration ± one standard error (error bar). Significant differences are indicated.

We further examined sera for 7 circulating cytokines in response to vaccination and oral challenge (Fig 4). No elevated levels of serum cytokines were detected in piglets at 0, 10, 21 and 28 days post vaccination. After oral challenge, we detected elevated levels of IFN-α in sera of control piglets at day 3 (p < 0.001), which coincided with higher fecal RV antigen shedding and diarrhea scores, but little elevation in sera of ID IRV or IM IRV piglets. Piglets in control group had a peak level of IL-8 three days post oral challenge, whereas piglets in ID IRV and IM IRV groups showed delayed IL-8 peak at day 5 following oral challenge.

### ID- and IM-administered IRV reduced fecal RV antigen shedding and diarrhea severity post-challenge

We evaluated the protection of ID- or IM-administered IRV against RV infection in Gn piglets challenged with VirWa RV by comparing RV antigen shedding in rectal swab samples (Table 1). All 3 piglets in the control group shed RV antigen with relative high OD values for a mean duration of 5.3 days. By contrast, only 1 of the 4 piglets in ID IRV group shed rotavirus antigen in reduced amount and with a mean duration of 0.75 day and a mean cumulative OD of 0.19, indicating a 91% decrease in antigen shedding. Similarly, only one animal in IM IRV group shed rotavirus antigen with a mean duration of 0.5 day and a mean cumulative OD of 0.07, indicating a 97% reduction in rotavirus shedding in this group.

**Table 1:** RV antigen shedding and diarrhea in Gn piglets post-challenge with VirHRV Wa.

		RV antigen shedding ^a^				Diarrhea ^b^			
Treatment Group	N	Animals with shedding (%)	Mean duration (days)	Mean cumulative OD	% Decrease in shedding	Animals with diarrhea (%)	Mean duration (days)	Mean cumulative score ^c^	% Decrease in cumulative score
Control	3	3/3 (100%)	5.3	2.05	-	2/3 (66%)	1.3	2.7	-
ID	4	1/4 (25%)	0.75	0.19	91%	0/4 (0%)	0	0	100%
IM	4	1/4 (25%)	0.5	0.07	97%	2/4 (50%)	0.5	1	63%
P-value ^d^	-	0.143	**0.026**	**<0.001**		0.143	0.068	0.078	
P-value ^e^		0.143	**0.016**	**<0.001**		1.000	0.415	0.445	

Gn piglets were vaccinated with control, ID and IM IRV and orally challenged with VirHRV Wa as described in the text. N = number of animals per group.

^a^ Determined by Premiere™ Rotaclone®.

^b^ Diarrhea duration was defined as the number of days with fecal score ≥2. Stool consistency was scored daily (0 = normal; 1 = pasty; 2 = semi-liquid [moderate diarrhea]; 3 = liquid [severe diarrhea]).

^c^ Mean cumulative score was ∑ (faecal scores ≥2)/number of animals.

^d^ Control vs ID.

^e^ Control vs IM.

We also evaluated the protection of ID- or IM-administered IRV against RV diarrhea in Wa RV-challenged Gn piglets by comparing diarrhea scores in vaccinated animals and placebo controls (Table 1). Two of the three piglets in control group had diarrhea score of 2 for a mean duration of 1.3 days and with a mean cumulative score of 2.7. By contrast, all 4 piglets in ID IRV group had scores ≤1, showing 100% decrease in diarrhea cumulative score as a group and complete protection against diarrhea. Two of the 4 piglets in IM IRV group had a score of 2 for only one day (post challenge day 1 or 2), while the other two had scores ≤1 for all 10 days, showing 63% reduction in diarrhea cumulative score as a group and at least partial protection against diarrhea.

## Discussion

We previously reported enhanced immunogenicity and apparent dose sparing of IRV coated on a microneedle patch when compared with IM-administered IRV without an adjuvant in mice. In this study we further evaluated the immunogenicity and protective efficacy of ID-administered IRV without adjuvant using a hollow microneedle compared with IM-administered IRV formulated with Al(OH)_3_ adjuvant. We demonstrated that both ID- and IM-administered IRV induced comparable IgA, IgG, IgG antibody avidity and neutralizing antibody responses, and conferred complete or at least partial protection against homologous rotavirus infection (challenge) and diarrhea in piglets. Of particular interest was the detection of high titers of IgA in sera of both ID and IM IRV-vaccinated animals, an observation that challenges the dogma that parenteral vaccination may not induce IgA response [39–41]. Whether serum IgA was transferred to the gut and together with serum IgG, mediated protection against rotavirus infection and diarrhea requires further investigations. Systemic routes of vaccination under some circumstances (antigen, adjuvant, and delivery vehicle) have the potential to induce immune responses in the systemic system and multiple mucosal compartments [42].

The findings in the present study agree with our previous report that IM-administered IRV was effective in inducing protective immunity against rotavirus infection in piglets [22]. Our results further showed that a parenterally administered IRV was effective against rotavirus diarrhea as well. However, two major differences merit comments here. We vaccinated animals with 5 μg antigen formulated with Al(OH)_3_ in the present study compared with 50 μg antigen formulated with Al(PO)_4_ in the prior study. These results indicate that Al(OH)_3_ or Al(PO)_4_ are effective adjuvants for IRV or even an IRV-containing combination vaccine. The observation that a 1/10 of the previously tested high-dose antigen was as effective in inducing protective immunity is promising for a low cost vaccine.

The skin is a dynamic organ that harbors elements of the innate and the adaptive immune systems. Skin is particularly rich in dendritic cells (Langerhans cells) in the epidermis which, when activated by infection, can mount a robust immune response. Skin immunization has been demonstrated to be effective against rabies, tuberculosis and other infectious diseases, including successful eradication of smallpox. However, those ID vaccines were delivered using old devices (i.e., bifurcated needle) and the Mantoux technique (regular needle and syringe) which were technically difficult and thus required highly trained personnel and can also be quite painful [43, 44]. The MicronJet600^®^ device used in this study was designed to be user friendly and requires minimum training. In a study that compared trivalent influenza vaccine (TIV) administered ID or IM in adults aged ≥ 65 years showed that ID administration was associated with decreased pain at the site of injection (P < 0.01) compared to the IM groups [45].

Interestingly in our study, IFN-α, which is an innate anti-viral cytokine, was not associated with protection but showed association with HRV replication as indicated by higher fecal shedding OD post challenge in the control group. Our findings line up with the observation in children, where vomiting episodes and RV diarrhea significantly correlated with IFN-α levels [46]. In animal studies, IFN-α administered before RV infections reduced RV diarrhea in pigs and calves [47, 48]. However, pretreatment with IFN-α in neonatal mice had no effect on virus shedding, suggesting species specific effects [49], and the role of IFN-α in RV clearance in mice may differ between homologous and heterologous RV infections [50]. In addition, chemokine IL-8 response was delayed by a few days in IRV-vaccinated piglets compared to controls, the significance of this finding is not clear.

The present study is subject to several implications and limitations. We simultaneously compare ID and IM routes for administering IRV. Our findings demonstrated that both ID IRV and IM IRV induced robust IgA response in sera of piglets suggesting that serum IgA antibodies may serve as a proxy to assess the immunogenicity of IRV in clinical trials, similar to the approach used for the development and licensure of oral rotavirus vaccines. Because of volume restriction, we chose two sites to administer the vaccine. For IRV vaccination in humans, we would anticipate a single application site similar to ID-administered concentrated Fluzone vaccine (Intanza^®^). The piglets were orally inoculated with a virulent human RV Wa G1P[8] at a dose of 5x10^5^ ID50, the identical dose reported in our previous study [22]. However, because of the cost and the size of piglets in each litter, we were not able to conduct dosing experiments to determine whether a further lower dose of IRV would be as effective to induce protection. In addition, we had to use small numbers of animals in each group. Nevertheless, the findings of this proof of concept study demonstrate the safety and protective efficacy of IRV via IM or ID administration in a large animal model. These safety and efficacy data in pre-clinical studies should help encourage clinical development of a standalone IM or ID IRV first and subsequently an IRV-containing combination vaccine suitable for use in infants. A safe and efficacious IRV combination vaccine has great potential to be implemented globally as it does not need a separate cold chain and thus simplifies administration, and particularly in developing countries where RV mortality is high and current oral vaccines seem less efficacious.

## Supporting Information

S1 File Ethics approval. (PDF)
