# Supplementary material for: Skin Vaccination against Rotavirus Using Microneedles: Proof of Concept in Gnotobiotic Piglets
Source: PLoS One. 2016 Nov 8;11(11):e0166038. doi: 10.1371/journal.pone.0166038 (PMC5100943; doi:10.1371/journal.pone.0166038)
Supplement: S1 File — (PDF) [file pone.0166038.s001.pdf]

Institutional Animal Care and Use Committee
Office of Responsible Research Practices
1960 Kenny Road
Columbus, OH 43210-1063

Phone (614) 292-4494
Fax (614) 688-0366
IACUCinfo@osu.edu
www.orrp.osu.edu

Letter of IACUC Protocol Approval

2010A00000088-R2
Immunization of Gnotobiotic Pigs with Candidate Rotavirus Vaccines (Renewal 2)
Linda Saif
OARDC Food Animal Health

Dear Dr. Saif:

The Institutional Animal Care and Use Committee (IACUC) has **APPROVED** the above referenced Animal Use Protocol (AUP).

Date of Approval: 3/03/2016
Date of Expiration: 3/03/2019

During this three year approval period, annual reviews are required. The protocol is due for a year one Annual Review no later than 3/03/2017, and a year two Annual Review no later than 3/03/2018.

The IACUC staff will make every effort to send the Principal Investigator annual reminders. However, the Principal Investigator is responsible for submitting an Annual Review in advance of the annual review due dates to ensure continuing IACUC approval. It is very important that these deadlines are not missed. Failure to submit an Annual Review on time will result in all persons listed under this protocol losing access to animal facilities and animal ordering, and may potentially result in the termination of the protocol.

To continue this research beyond the three year approval period, a new protocol submission will be required. To avoid a lapse in IACUC approval, it is essential that the completed renewal protocol be submitted and approved by the IACUC prior to its expiration date.

Federal regulations do not permit the IACUC to extend any approval periods. If a renewal protocol has not been processed and approved by the IACUC prior to 3/03/2019, IACUC approval for the work under the above referenced protocol will expire. Should IACUC approval expire, all activities involving the care and use of animals must cease immediately. Any activities conducted under the protocol after expiration will be in direct violation of federal regulations and institutional and IACUC policies.

It is the responsibility of the Principal Investigator to notify the IACUC of any proposed changes regarding the work described within this protocol. The Principal Investigator agrees that no such changes will be implemented until approved by the IACUC, except where absolutely necessary to eliminate apparent immediate hazards to person(s) and/or animal(s).

Please forward a copy of this document to your sponsored program officer. The sponsored program officer will certify this review for external sponsors.

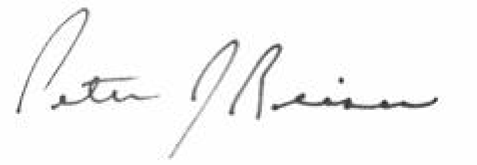

Peter J. Reiser, Ph.D.
IACUC Chair
